# Comparative Transcriptomics Uncovers Upstream Factors Regulating *BnFAD3* Expression and Affecting Linolenic Acid Biosynthesis in Yellow-Seeded Rapeseed (*Brassica napus* L.)

**DOI:** 10.3390/plants13060760

**Published:** 2024-03-07

**Authors:** Xiao-Yu Chen, Hao-Xue Wu, Xiao-Han Zhang, Rong-Hao Guo, Kang Li, Yong-Li Fu, Zhen Huang, Ai-Xia Xu, Jun-Gang Dong, Cheng-Yu Yu

**Affiliations:** College of Agronomy, Northwest A&F University, Taicheng Road 3, Yangling 712100, Chinahuang_zhen.8@163.com (Z.H.); xuaixia@nwsuaf.edu.cn (A.-X.X.);

**Keywords:** *Brassica napus*, linolenic acid, transparent testa, FAD2, FAD3, LEC1, ABI3, FUS3

## Abstract

α-Linolenic acid (ALA) is an important nutrient component in rapeseed oil, and rapeseed breeders want to either restrain or enhance the function of fatty acid desaturases (FADs) in the ALA biosynthesis pathway. To determine the reason for the upregulation of rapeseed *BnFAD* genes in two high-ALA accessions, R8Q10 and YH25005, we compared their transcriptome profiles in the seed at 24 days after pollination (DAP) with those of two low-ALA lines, A28 and SW. The expression levels of twenty-eight important genes in the seed samples at 20, 27, and 34 DAP were also investigated using an RT-qPCR. The expression levels of genes involved in flavonoid and proanthocyanidin synthesis, including *BnCHS*, *BnCHI*, *BnDFR*, *BnFLS1*, *BnLDOX*, *BnBAN*, *BnTT10*, and *BnTT12* and genes encoding the transcription factors BnTT1, BnTT2, BnTT8, and BnTT16 were lower in R8Q10 and YH25005 than in A28 and SW. The expression levels of genes encoding master transcription factors in embryo development, such as *BnLEC1*, *BnABI3*, *BnFUS3*, *BnL1L*, *BnAREB3*, and *BnbZIP67*, were elevated significantly in the two high-ALA accessions. Combined with previous results in the Arabidopsis and rapeseed literature, we speculated that the yellow-seededness genes could elevate the activity of BnLEC1, BnABI3, BnFUS3, and BnbZIP67, etc., by reducing the expression levels of several *transparent testa* homologs, resulting in *BnFAD3* and *BnFAD7* upregulation and the acceleration of ALA synthesis. Yellow-seededness is a favorable factor to promote ALA synthesis in the two high-ALA accessions with the yellow-seeded trait. These findings provide initial insights into the transcriptomic differences between high-/low-ALA germplasms and a theoretic basis for seed quality breeding.

## 1. Introduction

Oil rapeseed (*Brassica napus*. L. AACC, 2n = 38) is the second largest oil crop in the world. It provides great nutritional value to human beings, with over 93% unsaturated fatty acids, including oleic acid (C18:1, ω-9), linoleic acid (C18:2, ω-6), and α-linolenic acid (ALA, C18:3, ω-3), when the biosynthesis of erucic acid (C22:1) is blocked. A low ALA level was thought to be an important goal for rapeseed quality breeding owing to its higher smoking point and tolerance to oxidation [1,2]. Recently, high-ALA oil rapeseed has also been researched for its high nutritional value in omega-3 fatty acids [3,4] because ALA is an indispensable fatty acid to humans. ALA is the precursor of longer-chain omega-3 fatty acids, eicosapentaenoic acid and docosahexaenoic acid, which have beneficial effects on risk factors for cognitive impairment and cardiovascular diseases. Previous studies on the genetic control of rapeseed ALA content were based on plant accessions with low (ca. 3%) and moderate (ca. 9%) ALA contents [1,2], but no high-ALA genotype was involved [4]. Recently, several new germplasms of *B. napus* with high-ALA contents were created through various breeding methods including mutagenesis by exposure to gamma radiation [5], inter-varietal crossing [3], and directional selection in the yellow-seeded germplasm [4]. Two new *B. napus* germplasms, YH25005 and R8Q10, developed by us [4], contain 15% to 21% ALA and are useful gene donors to improve the quality of *B. napus*. Interestingly, we found that all the high-ALA progenies derived from YH25005 or R8Q10 crossed with other normal genotypes were yellow-seeded. Previous studies have shown that with the same genetic background, the yellow-seeded type often surpasses the brown-seeded type in its high oil content, as well as its high protein content and low cellulose content [6]. Therefore, an in-depth exploration of the genetic mechanism favoring ALA accumulation in the yellow-seeded germplasm is of significance to controlling ALA synthesis and improving seed quality.

The two-step biosynthesis of Arabidopsis seed oil ALA from oleic acid is directly catalyzed by chloroplast-located Fatty Acid Desaturase 2 (FAD2) and FAD3 or endoplasmic reticulum-located FAD6, FAD7, and FAD8. Defects in the *BnFAD2* or *BnFAD3* genes are the main reasons for a decrease in the ALA content and an increase in the oleic content in the seed [1,2]. Previous studies focused on the relationship between the exon variations of the *BnFAD2* and *BnFAD3* genes and low-ALA and high-oleic-acid traits [1,2]. Although such defects in the *BnFAD2* and *BnFAD3* genes were reported frequently, gain-of-function mutations in these genes are rarely found. Accordingly, the breeding target to reduce the ALA content in rapeseed is easier to achieve than the target to elevate ALA. The biosynthesis of unsaturated fatty acid is controlled by many genetic factors which form a complex network; therefore, it is necessary to find more genetic loci to finely control oleic acid and ALA synthesis [7]. For instance, the influence of mutations of the *BnFAD2* and *BnFAD3* promoters on their expression levels, which are regulated by some upstream transcription factors (TFs), is less understood. In the model plants Arabidopsis and soybean, the expression levels of *FAD2* and *FAD3* are regulated by some TFs, such as LAFL (collectively referring to LEC1, ABI3, FUS3, and LEC2) or LAZA (LEC1, AREB3/bZIP66, bZIP67, and ABI3) members, which regulate embryo development and the accumulation of storage substances [8]. Moreover, previous results showed that in some Arabidopsis *transparent testa* (*tt*) mutants, including *tt16*, *tt2*, *tt1*, and *tt8*, the expression levels of *FUS3*, *LEC1*, *ABI3*, and *FAD3* were upregulated [9,10,11,12].

However, the transcriptional regulation of the *FAD2* and *FAD3* homologous genes in tetraploid rapeseed and their effect on fatty acid biosynthesis and accumulation are more complex than in the model plants. The *B. napus* genome harbors three or four *FAD2* and six *FAD3* homologous copies, and their promoter sequences are obviously differentiated. Their expression patterns in seed development may be quite different [13]. It is uncertain whether rapeseed embryo master regulators of LAFL/LAZA members actually regulate the expression of *BnFAD2*, as predicted in Arabidopsis and soybean [8]. The inheritance of ALA content follows a quantitative genetic model of the mixed effect of two major loci plus minor-effect polygenes [4]. In light of the difficulties in exact QTL mapping in the tetraploid genome, which is often based on the construction of a recombinant inbred line or double haploid population, we first aim to determine the expression regulation of *BnFAD2* and *BnFAD3*. The expression levels of most *BnFAD2* and *BnFAD3* copies in the high-ALA accessions were much higher than those in wildtype ZS11 and much higher than in the two low-ALA genotypes, A28 and SW [13]. Some important SNP or InDel mutation sites were found in the promoters of high-, moderate-, and low-ALA accessions [13]. Thus, their binding situation by upstream TFs may be different. It is necessary to study the regulation profile of master TFs on the expression of *BnFAD2* and *BnFAD3* genes. However, no global transcriptional regulation in a high-ALA germplasm has been reported to date, and there is a knowledge gap in the transcriptome information of high-ALA rapeseed. Comparative transcriptomics is a powerful tool to comprehensively characterize the molecular basis of tissue development, and it provides a solid foundation for achieving a mechanistic understanding of many biological processes. Therefore, the expression levels of genes related to ALA synthesis and the flavonoid synthesis pathway were investigated in this study using a transcriptomic analysis combined with real-time quantitative PCR (RT-qPCR) detection.

## 2. Results

### 2.1. Screening Key Differentially Expressed Genes during Seed Development

**Figure 1:**
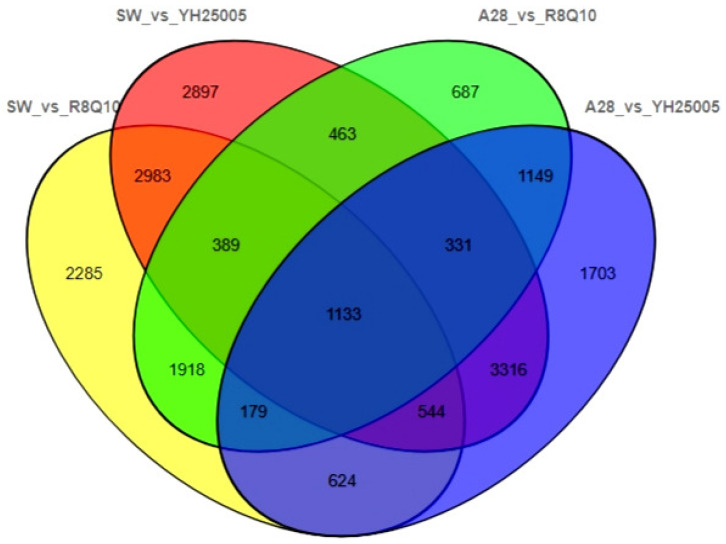
Venn diagram showing the number of DEGs shared by the four sets of transcriptomes.

To uncover the transcriptional regulation profile during seed fatty acid synthesis, we compared the transcriptomic data (deposited in NCBI GEO database under accession No. GSE186952) of young seeds at 24 DAP (in the early-mid stage of seed development) and found a total of 6249 to 12056 DEGs (Table 1) in a pairwise comparison between A28 (or SW) and R8Q10 and A28 (or SW) and YH25005 (Supplementary Materials, Dataset S1). As shown in a Venn diagram (Figure 1), there were 1133 DEGs shared by the four sets in the transcriptome comparison. There were 5324 DEGs shared by A28 and SW when they were compared with YH25005, and there were 3619 DEGs when they were compared with R8Q10, indicating more complex transcriptional regulation in YH25005.

The DEGs related to lipid synthesis, transport, and metabolism are shown in four heatmaps (Supplementary file Figure S1), and some TFs affecting seed development are listed in Table 2. Eight protein–protein interaction networks were predicted (Supplementary file Figure S2) based on the Arabidopsis homologous proteins encoded by the up- or downregulated DEGs. Some important DEGs in R8Q10, compared with SW or A28, included *BnTT8*, *BnTT12*, *BnLEC1*, *BnFUS3*, *BnLEC2*, and *BnAREB3* (*ABI5-like2*) (Table 2). Similarly, the DEGs in YH25005, compared with SW or A28, included *BnTT1*, *BnTT16*, *BnTT12*, *BnTT9*, and *BnABI3*.

Several *BnFAD3* copies (*BnaA03g13590D*, *BnaA04g17150D*, *BnaA05g12360D*, *BnaC04g14820D*, and *BnaC04g40760D*) were in the upregulated DEGs of A28 vs. YH25005 and SW vs. YH25005, which is consistent with previous results [13]. A *BnFAD6* copy, *BnaA05g15670D,* was downregulated in A28 vs. R8Q10 and A28 vs. YH25005, but another copy, *BnaC01g07470D,* was upregulated in SW vs. R8Q10. Two *BnFAD7* copies (*newgene_551* and *newgene_11478*) were downregulated in the comparison of A28 vs. R8Q10 and SW vs. R8Q10, but *BnaA03g31600D* and *BnaA05g27840D* were upregulated in A28 vs. YH25005 and SW vs. YH25005. However, chloroplastic *FAD6/FAD7* has a minor effect on seed oil accumulation; hence, the contribution of a few copies of *BnFAD6/BnFAD7* may be masked by the complementary effect of other copies. The genes encoding BnFUS3, BnNF-YC, and other TFs were upregulated in R8Q10 and YH25005. The differential expression of these TFs may affect *BnFAD3* expression. In addition, several genes related to the fatty acid metabolism pathway, including *BnACP*, *BnACBP*, and *BnfatB*, also appeared frequently as DEGs. The abovementioned DEGs correspond to a protein interaction network involving fatty acid metabolism and related regulation (Supplementary Materials, Figure S2).

### 2.2. KEGG Pathway Enrichment Analysis

**Figure 2:**
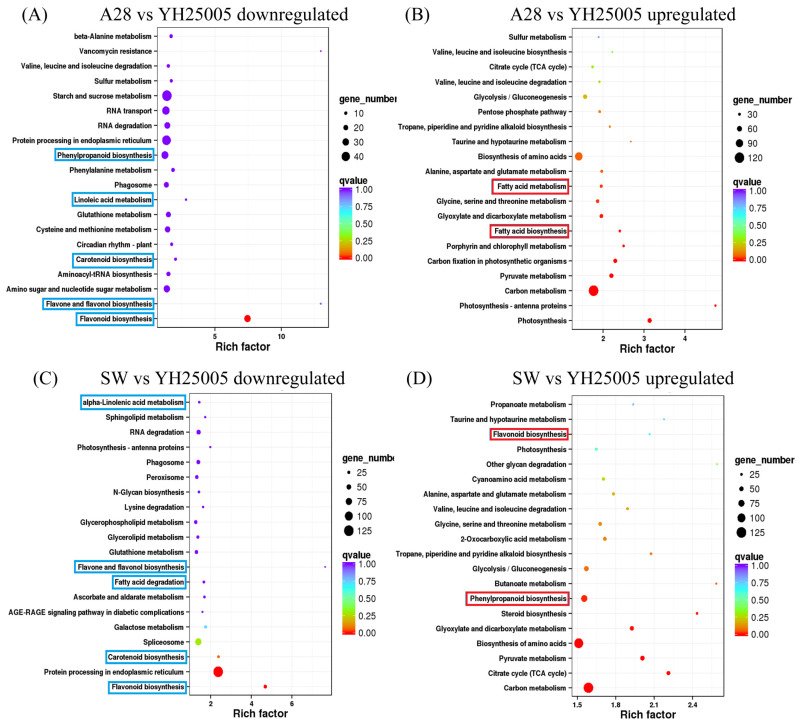
Enrichment of KEGG pathways based on up/downregulated DEGs of YH25005 in comparison to SW and A28. Some interesting KEGGs related to fatty acid accumulation and seed coat pigments are indicated by blue boxes (downregulated DEGs) or red boxes (upregulated).

**Figure 3:**
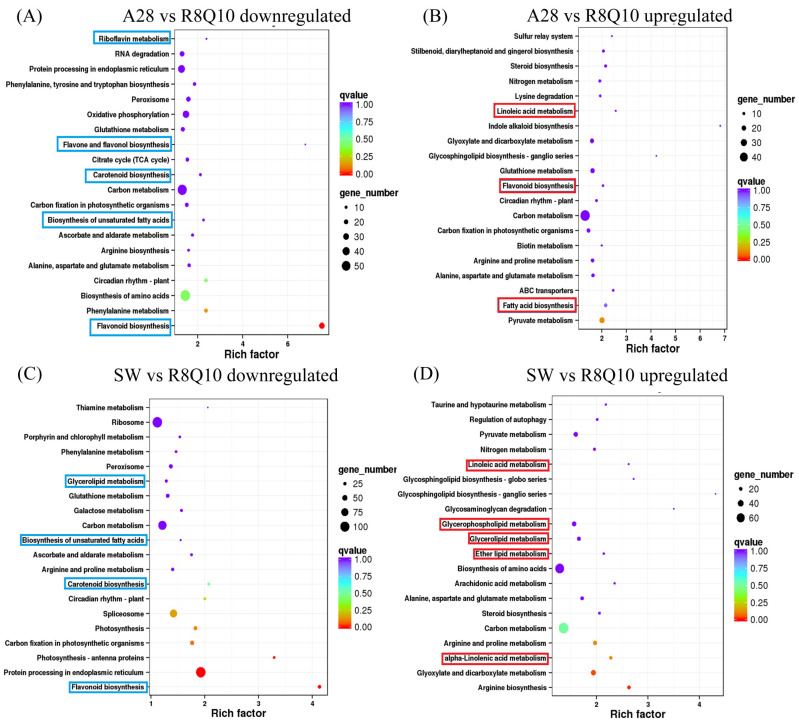
Enrichment of KEGG pathways based on up/downregulated DEGs of R8Q10 in comparison to SW and A28. Some interesting KEGGs related to fatty acid accumulation and seed coat pigments are indicated by blue boxes (downregulated DEGs) or red boxes (upregulated).

Among the top enriched KEGG pathways, the biosynthesis of flavonoid, flavone, and flavonol, carotenoid, and phenylpropanoid, unsaturated fatty acid biosynthesis, and fatty acid degradation were significantly downregulated pathways. The flavonoid biosynthesis pathway often has the largest enrichment factor and the lowest Q-value (Figure 2 and Figure 3), indicating the importance of flavonoid biosynthesis among the four pairwise comparisons. Among the upregulated KEGG pathways, fatty acid metabolism and linolic acid metabolism were frequently significant. Both high-ALA and yellow-seededness traits are a signature of R8Q10 and YH25005 in comparison to A28 and SW, indicating a possible association between yellow-seededness (or transparent testa) and unsaturated fatty acid biosynthesis, as previous results suggested [9,10,11,12,13,14,15]. In addition to the flavonoid biosynthesis pathway, pathways such as glutathione metabolism, carbon metabolism, glyoxylate and dicarboxylate metabolism, and pyruvate metabolism are also significant pathways in the KEGG enrichment for the four pairwise comparisons, but their relationships with ALA accumulation are unknown.

### 2.3. Expression Level of Rapeseed FAD Genes in Different Seed Development Stages

**Figure 4:**
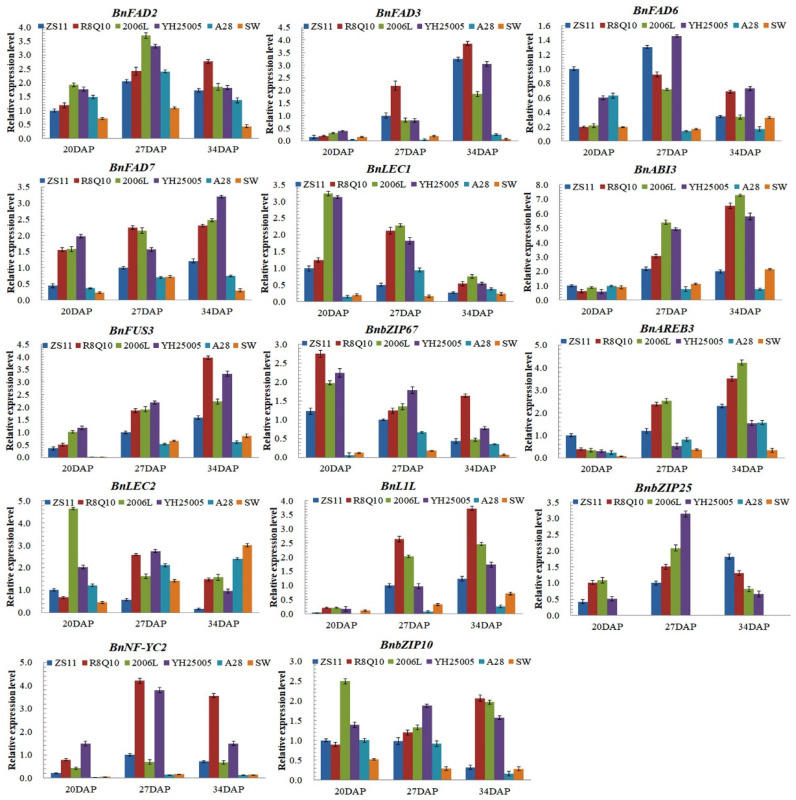
Expression levels of *BnFAD* genes and eight genes encoding embryo master TFs in the six rapeseed genotypes with different ALA contents.

In the transcriptome analysis, it was found that the expression of genes encoding proteins important in ALA synthesis and upstream TFs were upregulated in high-ALA accessions. Thus, some interesting genes were selected for an RT-qPCR to verify their expression differences in seeds 20, 27, and 34 days after the simultaneous pollination of 2006L, R8Q10, YH25005, ZS11, A28, and SW (Figure 4). The expression levels of *BnFAD2* and *BnFAD3* in the three high-ALA rapeseeds were significantly higher than those of ZS11, SW, and A28. The expression level of the *BnFAD7* gene in the high-ALA accession was higher than that in ZS11 and much higher than that in the low-ALA accession, especially at 34 DAP. It has not been confirmed whether the upregulation of the expression of the plastid-located BnFAD7 in seeds affects seed ALA accumulation. The expression of *BnFAD6* in SW was lower than that in A28 and much lower than in the other accessions, which indicates that *BnFAD6* might also affect the content of oleic acid to some extent.

### 2.4. Differential Expression of TFs Related to ALA Synthesis

The expression levels of *BnFUS3*, *BnABI3*, *BnL1L*, and *BnAREB3* in different accessions increased gradually along the three developing stages, while the expression of *BnLEC1* and *BnbZIP67* decreased slightly (Figure 4). The expression of *BnNF-YC2* in 2006L and YH25005 was significantly upregulated in the middle stage (27 DAP), but *BnABI3* and *BnFUS3* were more expressed in the mid-late stage (34 DAP) of the embryonic development of the plant seeds.

The expression levels of *BnFUS3*, *BnABI3*, *BnLEC1*, *BnL1L*, *BnbZIP67*, *BnbZIP25*, and *BnbZIP10* in the three high-ALA accessions were higher than that in ZS11 and much higher than those in the low-ALA accessions. Particularly, the expression of *BnbZIP10* in SW was lower than in A28 and ZS11. The expression of *AREB3/bZIP66* in 2006L and R8Q10 was much higher than that in the other accessions, especially in the middle and mid-late stages. The upregulated expression of genes encoding LAFL members in high-ALA accession may elevate *BnFAD3* (possibly also including *BnFAD2* and *Bn FAD7*) expression.

### 2.5. Expression Levels of Rapeseed Genes Related to Flavonoid Synthesis

**Figure 5:**
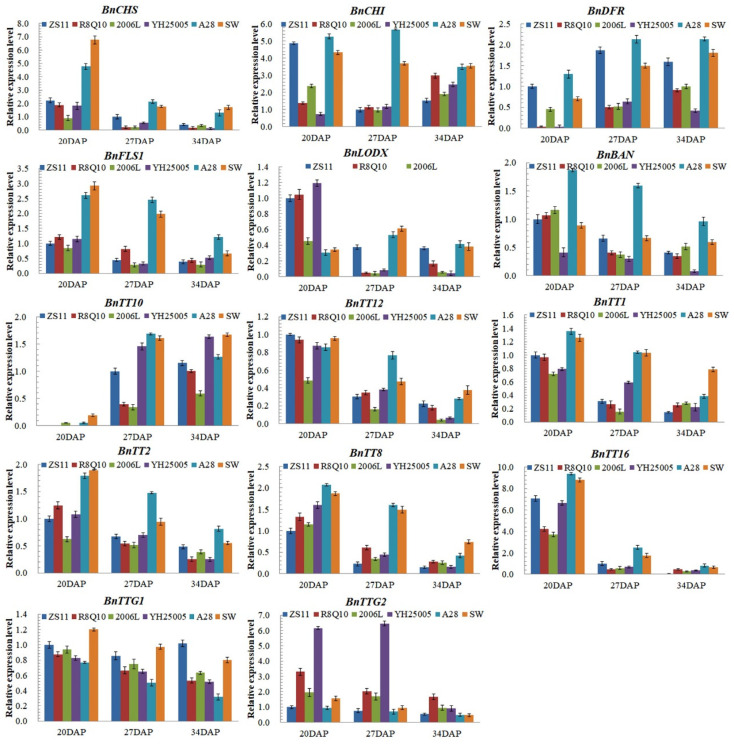
Expression levels of important *TT* genes in the six rapeseed genotypes with different ALA contents.

*TT4/CHS*, *TT5/CHI*, *FLS1/FLS*, *TT7/DFR*, *TT18/LODX*, *BAN/ANR*, and *TT10* are all structural genes in the flavonoid synthesis pathway. In the brown-seeded plant accessions ZS11, A28, and SW, the expression of these structural genes and the transporter *BnTT12* decreased gradually with seed development, while *BnTT10* increased gradually with seed development (Figure 5). This trend corresponds well to the fact that the synthesis of flavonoids mainly occurs in the early stage of seed development; then, in the medium and later stages, TT10’s important function is to condensate proanthocyanidins from flavonoids and deepen the seed coat color gradually in the mid-late stage. The expression levels of *BnCHS*, *BnCHI*, *BnDFR*, *BnFLS*, *BnLDOX*, and *BnBAN* in the low-ALA accessions were much higher than those in the three high-ALA accessions. The expression of *BnTT10* in the high-ALA accessions was lower than in ZS11 and significantly lower than in the low-ALA accessions. The expression levels of *BnTT12* in the low-ALA accessions A28 and SW were significantly higher than in other accessions in the middle and mid-late stages.

Like in Arabidopsis, rapeseed *TT1*, *TT2*, *TT8*, *TT16*, *TTG1*, and *TTG2* encode important TFs in the flavonoid and proanthocyanidin synthesis pathways and mainly regulate the synthesis and metabolism of flavonoids by regulating the expression of structural genes and transporter genes. The expression of their rapeseed homologous genes gradually decreased with the development of seeds (Figure 5), which is consistent with the expression trends of most of the above-mentioned structural genes. The expression levels in ZS11 and the high-ALA accessions were not significantly different. However, the expression levels of *BnTT1*, *BnTT2*, *BnTT8*, and *BnTT16* in the two other low-ALA accessions were higher than those in ZS11 and the high-ALA accessions. Lowered expression levels of the four genes would reduce the seed flavonoid content to a certain extent; thus, it is possible that the ZS11 genome may also harbor the yellow-seededness gene but less than R8Q10 and YH25005, although ZS11 has a light brown seed coat rather than a visible yellow-seed phenotype. Unexpectedly, the expression of *BnTTG2* in the three high-ALA accessions was much higher than in ZS11 and the two low-ALA accessions.

## 3. Discussion

### 3.1. Upstream TFs Affect the Expression Levels of FAD Genes 

It was shown that Arabidopsis ABI3, FUS3, and bZIP25 can indirectly adjust *FAD3* expression through bZIP67 binding to the G-box motif in the *FAD3* promoter [16]. The biosynthesis of oleic acid was upregulated in a *fus3* mutant [17]. A sesame bHLH protein can bind the E-box or G-box motif in a *SeFAD2* promoter and regulate its expression [18]. Using chromatin immunoprecipitation technology, it was supposed that Arabidopsis ABI3 could directly target and regulate *FAD3* and *FAD2* [19], and some soybean LAZA members shared many regulatory targets including *GmFAD2* and *GmFAD6* [8]. To our knowledge, which LAZA/LAFL member targets *FAD6/FAD7* has not been validated yet. We previously identified some sequence mutations in the promoter regions of different copies of *BnFAD* genes in the six accessions used in the present study [13]. These mutations may affect their expression levels during LAFL/LAZA member binding on some cis-regulatory elements. Therefore, uncovering the transcriptional regulation profile will be important to understanding the inheritance of high-ALA traits.

### 3.2. Some tt or Yellow-Seededness Genes Can Regulate LAFL or LAZA TFs

In mutants of Arabidopsis *TT* genes or their rapeseed homologous genes, most genes that participate in flavonoid biosynthesis will be downregulated, as shown in the literature [11,14,20,21,22], for example, *TT16*, *CHS/TT4*, *CFI/TT5*, *F3H/TT6*, *DFR/TT3*, *LDOX/TT18*, *ANR/BAN*, *TT12*, and *GSTF12/TT19* [14], resulting in significant changes in embryo development and oil accumulation. Until now, among the identified *TT*s, six genes, *TT1/WIP1*, *TT2/MYB123*, *TT8/bHLH042*, *TT16/AGL32*, *TTG1* (*WD40*), and *TTG2* (*WRKY*), encode TFs. An increasing number of results have suggested that some genes in the flavonoid biosynthesis pathway, especially the six genes encoding TFs, can affect the expression of LAFL/LAZA members. For example, FUS3, LEC1, etc., were activated in *tt2*, *tt8*, and *ttg1* mutants [10,11,12]. There is a negative correlation between the amount of proanthocyanidins in the seed coat and the fatty acid content in the embryo [10]. Arabidopsis *TT2,* expressed in both the seed coat and the embryo, and its protein can directly bind to the regulatory region of *FUS3* [10]. Arabidopsis *TT8* can directly target *LEC1*, *LEC2*, and *FUS3* [12]. Lian et al. [14] found that *BnABI3*, *BnLEC1*, and *BnL1L* were upregulated in transgenic seeds with the silencing of *BnTT1* using RNAi technology. The *BnFAD2* expression level increased in some strains of *Bntt8*-knockout mutants [23]. It would be interesting to determine how many *TT* members can affect the expression levels of genes encoding LAFL or LAZA members directly or indirectly.

### 3.3. Relationship between Yellow-Seededness and High-ALA Traits

Our previous results showed that YH25005 carries the yellow-seededness characteristic with different origins and a different genetic behavior than R8Q10 [4,23]. After an analysis of the seed quality parameters of 1594 breeding materials with various origins of seed color genes [24], it was found that there was a strong correlation between seed color and the contents of the three main fatty acids, and yellow-seeded lines often had higher ALA contents than brown-seeded ones (Table 3). This implies that yellow-seededness traits play a certain role in promoting ALA accumulation

In the present transcriptomic comparison and RT-qPCR analysis, *BnLEC1*, *BnAREB3*, *BnABI3*, *BnbZIP67*, *BnFUS3*, *BnbZIP25*, *BnFAD2*, *BnFAD3*, *BnFAD7*, etc., were generally upregulated compared to the low-ALA plants. Combined with the above evidence, this implies that yellow-seededness in both YH25005 and R8Q10 may be an important factor for increasing ALA synthesis indirectly through LAFL, LAZA, and other TFs. Two recent studies [25,26] found that transgenic plants with a fusion *FAD2-FAD3* gene from perilla (*Perilla frutescens*) and chia (*Salvia hispanica*) resulted in very high ALA levels of up to 37% and 39% in rapeseed oil. Meanwhile, structural genes in fatty acid biosynthesis including *BnACCD*, *BnFATA*, *BnSAD*, *BnSCD*, *BnDGAT1*, *BnDGAT2*, and *BnDGAT3* and the positive regulators *BnWRI1*, *BnLEC1*, *BnL1L*, *BnLEC2*, *BnABI3*, *BnbZIP67*, and *BnMYB96* were upregulated, while the oppositive regulators *BnTT1*, *BnTT2*, *BnTT8*, *BnTT16*, *BnTTG1*, and *BnTTG2* were downregulated [25,26]. This also supports our assumption that there is a connection between the downregulation of *TTs*, the upregulation of genes in LAFL members, and the activity elevation of BnFAD3.

**Figure 6:**
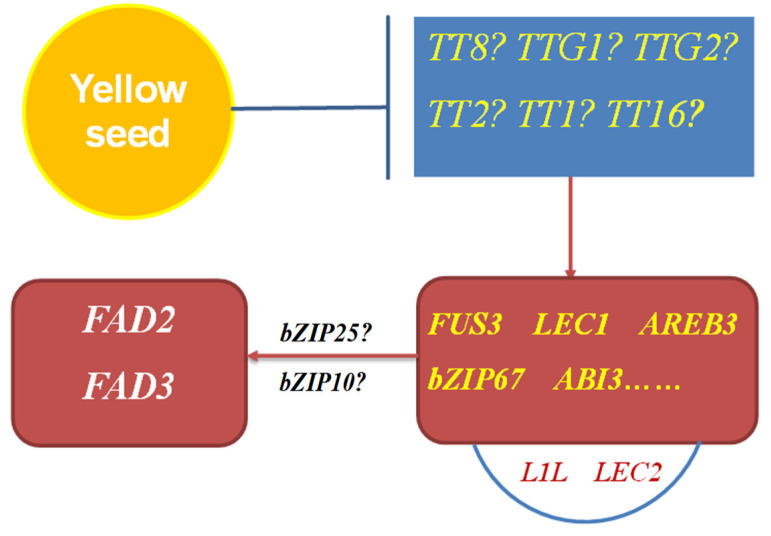
Scheme of the indirect regulation of *BnFAD2* and *BnFAD3* expression in yellow-seeded rapeseed.

Based on the above analysis, we proposed a regulatory network of *BnFAD3* expression in yellow-seeded rapeseed (Figure 6). The dysfunction of a *TT* gene, such as Arabidopsis *TT16*, *TT8*, and *TT2*, or rapeseed yellow-seeded genes, will cause the downregulation of most *TT* genes [14,20,21,22], like our present data in yellow-seeded accessions R8Q10 and YH25005. It is reasonable to suppose that downregulation of many *TT* genes will activate TFs such as LAFL or LAZA and then indirectly boost the enzyme activity of BnFAD2 and BnFAD3 (Figure 6). In other words, the yellow-seededness trait is a key positive factor that indirectly promotes the upregulation of *BnFAD3* (*BnFAD2*, *BnFAD6*, and *BnFAD7* may also be included) through a ‘bridge’ of LAFL/LAZA members. This knowledge is useful for a seed-quality breeding scheme, and the yellow-seededness trait can be used to increase the ALA content.

Nevertheless, there are some potential limitations of this study, such as its reliance on transcriptomic data and the need for further validation of the regulatory mechanisms identified. Cloning of the yellow-seededness gene in R8Q10 and YH25005 will help to establish a regulatory pathway model of *BnFAD2* and *BnFAD3* in those high-ALA and yellow-seeded plants. One of the obstacles to the study of yellow-seededness traits in rapeseed is that most genes that participate in flavonoid biosynthesis are synchronously downregulated, as in the Arabidopsis *tt2*, *tt8*, and *ttg1* mutants and the Brassica yellow-seeded lines [14,20,21,22]. This hinders screening for a candidate yellow-seededness gene through transcriptomic or metabolomic data using the reverse genetics strategy. Unfortunately, the strategy of map-based cloning yellow-seededness is not easily conducted due to the complexity of the tetraploid genome in *B. napus*. The map-based cloning of yellow-seededness genes was more successful in the diploid species *B. rapa*, such as the *ttg1*, *tt8*, and *tt1* homologous mutations [27,28,29,30]; however, to date, only one *BnA09MYB47a* mutation has been found to control the yellow-seededness character in the *B. napus* line GH06 [31].

## 4. Materials and Methods

### 4.1. Materials

Four plant accessions [13], two high-ALA genotypes, yellow-seeded R8Q10 (containing 20.11% ALA) and YH25005 (15.20%), and two low-ALA lines, brown-seeded A28 (3.34%) and SW (3.46% ALA and 86.32% oleic acid), were used in a transcriptomic comparison. Another yellow-seeded accession named 2006L with a high-ALA trait (18.16%) and an elite cultivar ZS11 (9.88%) were also included in the detection of gene expression.

### 4.2. Methods

#### 4.2.1. Transcriptomic Comparison among R8Q10, YH25005, A28, and SW Seeds

The R8Q10, YH25005, A28, and SW plants were grown in an experimental field of the Northwest A&F University. When they were flowering in the spring, flowers at same developmental stage were bagged for self-pollination. The seeds in the pods of plants 24 days after pollination (DAP) were collected, placed into liquid nitrogen, and then stored at −80 °C. Each accession had three independent biological replicates. The cDNA libraries for the seed samples were constructed by the Biomarker Technologies Corporation (Beijing, China), following the standard protocol of the NEBNext^®^ Ultra^TM^ RNA Library Prep Kit for Illumina (NEB, Ipswich, MA, USA) and the manufacturer’s recommendations. The cDNA libraries were sequenced on an Illumina Novaseq6000 platform with a PE150 flow cell type. After conducting a quality control check with the Q30 base percentage of all products higher than 95.43%, 84.19 Gb of clean data was obtained. The clean reads were mapped against the reference genome of Darmor-bzh (http://www.genoscope.cns.fr/brassicanapus/data/, accessed on 12 August 2019) using HISAT2 (https://cloud.biohpc.swmed.edu/index.php/s/hisat2-220-source/download, accessed on 12 August 2019). The transcripts were assembled using StringTie (https://ccb.jhu.edu/software/stringtie, accessed on 13 August 2019) through the maximum flow algorithm. The Fragments Per Kilobase of Transcription Per Million Fragments Mapped (FPKM) was used as an index to measure the level of gene expression. Differentially expressed genes (DEGs) between each pair of samples were screened using the DEseq2 (https://bioconductor.org/packages/release/bioc/html/DESeq2.html, accessed on 14 August 2019). A fold change (FC) ≥ 2 and a false discovery rate < 0.01 were used as screening criteria to identify differentially expressed genes. The DEGs were identified and annotated in various public databases. KEGG (Kyoto Encyclopedia of Genes and Genomes, https://www.genome.jp/kegg, accessed on 15 August 2019) pathways were enriched by using the hypergeometric test over the whole genome background. The above analysis was performed using the tool package on the BMKCloud platform (www.biocloud.net, accessed on 12 August 2019). Based on Arabidopsis data in the STRING database (https://string-db.org/, accessed on 15 March 2020), the possible interaction among proteins encoded by these DEGs was predicted.

#### 4.2.2. Detection of Gene Expression Levels by Real-Time Quantitative PCR (RT-qPCR)

Based on a comparative analysis of the four transcriptomes, some interesting genes (Table S1) were selected to validate their expression levels using an RT-qPCR with primers targeting the gene-conserved region. The rapeseed *beta-actin7* gene was used as an internal reference. Apart from the four plant accessions, another yellow-seeded accession, 2006L, and an elite cultivar, ZS11, were also included. The immature seeds at 20, 27, and 34 DAP, representing the early, middle, and mid-late stages of seed development, were used for RNA extraction in light of the importance of the period three to five weeks after pollination during the process of seed development and lipid accumulation in rapeseed [13,22]. The total RNA in the seed samples of these plant accessions, each in three replicates, was extracted using an RNA Isolation Kit (Omega Bio-tek Inc., Norcross, GA, USA) and then transcribed into cDNA. Quantitative PCR reactions were completed on a QUANTT Studio 7 flex PCR thermal cycler (ABI, Los Angeles, CA, USA). The reaction system contained Novoprotein SYBR Green qPCR Supermix plus (Novoprotein, Shanghai, China), Rox II, primers, cDNA sample, and RNase-free water.

## 5. Conclusions

We hypothesized that the yellow-seededness trait was a favorable factor significantly promoting ALA accumulation in the progenies of three yellow-seeded accessions. The loss of function of yellow-seededness genes can upregulate some master TFs in LAFL (LEC1, ABI3, FUS3, and LEC2) or LAZA (LEC1, AREB3, bZIP67, and ABI3) members, and these TFs will activate *BnFAD3* expression. This pathway makes an important contribution to the precise regulation and breeding of high-oleic-acid or high-ALA rapeseed. However, we should not assume that yellow-seededness is necessary for the high-ALA trait since other studies [3,5] also found some high-ALA accessions with a brown seed color. These uncertainties, such as how a specific yellow-seededness gene affects LAFL/LAZA members and how many TFs like LAFL/LAZA members can bind *BnFAD2*, *BnFAD3*, or *BnFAD7* promoters, need to be further studied. This study contributes to a better understanding of the transcriptomic differences between high/low-ALA rapeseed germplasms and provides a theoretical basis to genetically control the ALA content in seed-quality breeding.

## Figures and Tables

**Table 1:** Number of DEGs in a pairwise comparison between high- and low-ALA accessions.

Comparison	DEGs	Upregulated Genes	Downregulated Genes	GO Database	KEGG Database	NR Database
A28 vs. R8Q10	6249	3165	3084	5136	2414	6113
A28 vs. YH25005	8979	5438	3541	7535	3310	8780
SW vs. R8Q10	10,055	4581	5474	8457	3971	9817
SW vs. YH25005	12,056	6192	5864	10,154	4588	11,775
R8Q10 vs. YH25005	11,587	4909	6678	10,010	4675	11,341

**Table 2:** Representative DEGs encoding important transcription factors and transparent testa proteins in the transcriptomic comparison between high- and low-ALA genotypes.

R8Q10 Compared to SW	Log_2_(Foldchange)	R8Q10 Compared to A28	Log_2_(Foldchange)
*LEC1 NF-YB9 BnaAnng04140D*	1.467	*NF-YC3*	1.19
*bZIP38*, *AREB4 ABI5-like protein 7*	Inf	*bZIP25 BnaA06g16690D*	Inf
*AREB3 bZIP66 ABI5-like protein 2*	Inf	*bZIP10 BnaC09g00090D*	3.16
*AREB3 bZIP66 ABI5-like protein 2*	3.403	*bZIP46 BnaCnng20400D*	3.10
*bZIP67 ABI5-like protein 1*, *DPBF2*	2.9	*BnaA.TT10b*	2.23
*bZIP25*	2.358	*LEC1 BnaAnng04140D*	−1.43
*Laccase-15-like precursor*	3.89	*TRANSPARENT TESTA 12*	−1.84
*Laccase type BnaA.TT10b*	3.083	*TRANSPARENT TESTA 12-like*	−2.76
*bZIP46 BnaAnng26550D*	−1.65	*TRANSPARENT TESTA 12-like DTX 27*	−Inf
*TT8-like*	−1.39	*BnaC.TT8*	−1.80
*BnaC.TT8*	−1.67	*TT8-like*	−1.88
*TRANSPARENT TESTA 12*	−1.81	*AHA10*	−1.79
YH25005 compared to SW	Log_2_(Foldchange)	YH25005 compared to A28	Log_2_(Foldchange)
*bZIP38*, *AREB4*, *ABI5-like protein 7*	Inf	*ABI3*, *BnaC03g44820D*	1.60
*bZIP BnaCnng04010D*	3.648	*FUS3*, *BnaA02g28280D*	1.29
*bZIP25*, *BnaAnng30260D*	3.131	*FUS3*, *BnaC02g36350D*	1.36
*ABI5-like protein 3*, *bZIP12*, *DPBF4*	2.655	*FUS3*	1.64
*bZIP10*, *BnaA09g00980D*	1.962	*ABI3*, *BnaC03g44820D*	1.60
*bZIP35*, *AREB1 ABI5-like protein 4*	1.808	*L1L*, *NF-YB6*, *BnaC02g33430D*	3.73
*ABI3*, *BnaC03g44820D*	1.033	*NF-YA9*, *BnaA05g19990D*	1.56
*TRANSPARENT TESTA 12*	−2.68	*bZIP12*, *DPBF4*, *ABI5-like protein 3*	2.67
*TRANSPARENT TESTA 9*	−1.63	*bZIP25*, *BnaAnng30260D*	2.00
*TRANSPARENT TESTA 16*	−1.77	*TRANSPARENT TESTA 12*	−2.63
*TRANSPARENT TESTA 1*	−2.95	*TRANSPARENT TESTA 16*	−1.63

**Table 3:** Comparison of seed quality between 441 yellow-seeded lines and 1153 brown-seeded lines in our breeding materials, #.

Parameters	Seed Color	Average	Standard Error	*p*-Value of *T*-Test
Oil content %	Yellow	45.32	3.263	0.005
	Brown	43.41	3.756	
Oleic acid content %	Yellow	64.54	5.87	0.000
	Brown	66.86	6.112	
Linoleic acid content %	Yellow	19.03	2.352	0.000
	Brown	17.63	2.004	
Linolenic acid content %	Yellow	13.24	2.271	0.000
	Brown	10.98	1.514	

#: seed quality parameters were detected by a near-infrared reflectance spectroscope, a NIRSystem 5000 (FOSS, Hillerod, Denmark).

## Data Availability

All data generated or analyzed during this study are contained in the paper and in additional files in the Supplementary Materials.

## Supplementary Materials

The following supporting information can be downloaded at https://www.mdpi.com/article/10.3390/plants13060760/s1, Dataset S1. List of all DEGs in four transcriptomes. Table S1. The primer sequences designed for the rapeseed genes used in the RT-qPCR. Figure S1. Heatmap of the selected genes related to lipid biosynthesis, transport, and metabolism. Figure S2. Protein interaction network predicted based on DEGs.
