# Supplementary material for: Comparative Transcriptomics Uncovers Upstream Factors Regulating BnFAD3 Expression and Affecting Linolenic Acid Biosynthesis in Yellow-Seeded Rapeseed (Brassica napus L.)
Source: Plants (Basel). 2024 Mar 7;13(6):760. doi: 10.3390/plants13060760 (PMC10974354; doi:10.3390/plants13060760)
Supplement: Supplementary file 1 [file plants-13-00760-s001.zip › Figure S1. Heatmap of the selected DEGs related to lipid.pptx]

## Slide 1
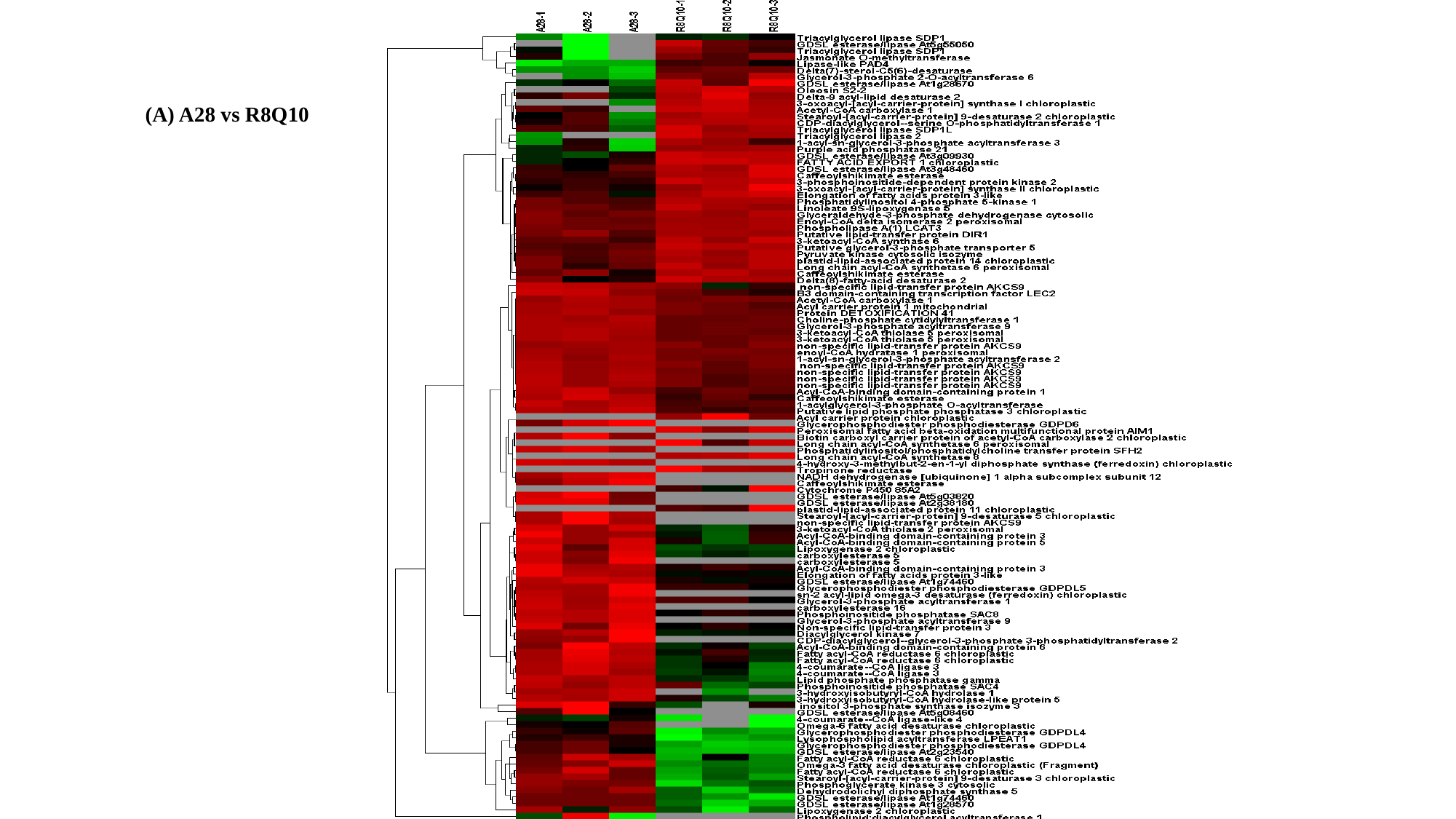

(A) A28 vs R8Q10

## Slide 2
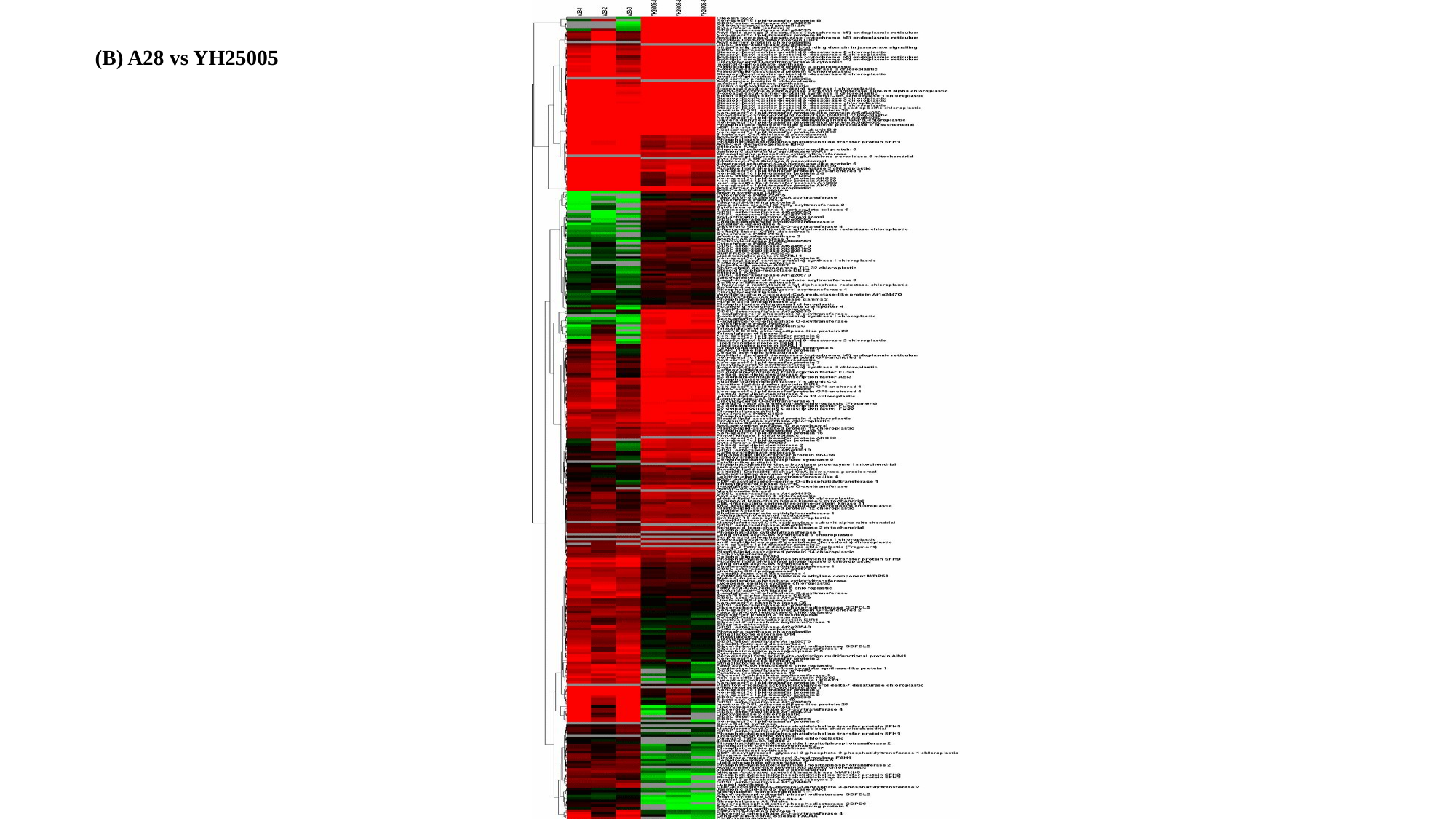

(B) A28 vs YH25005

## Slide 3
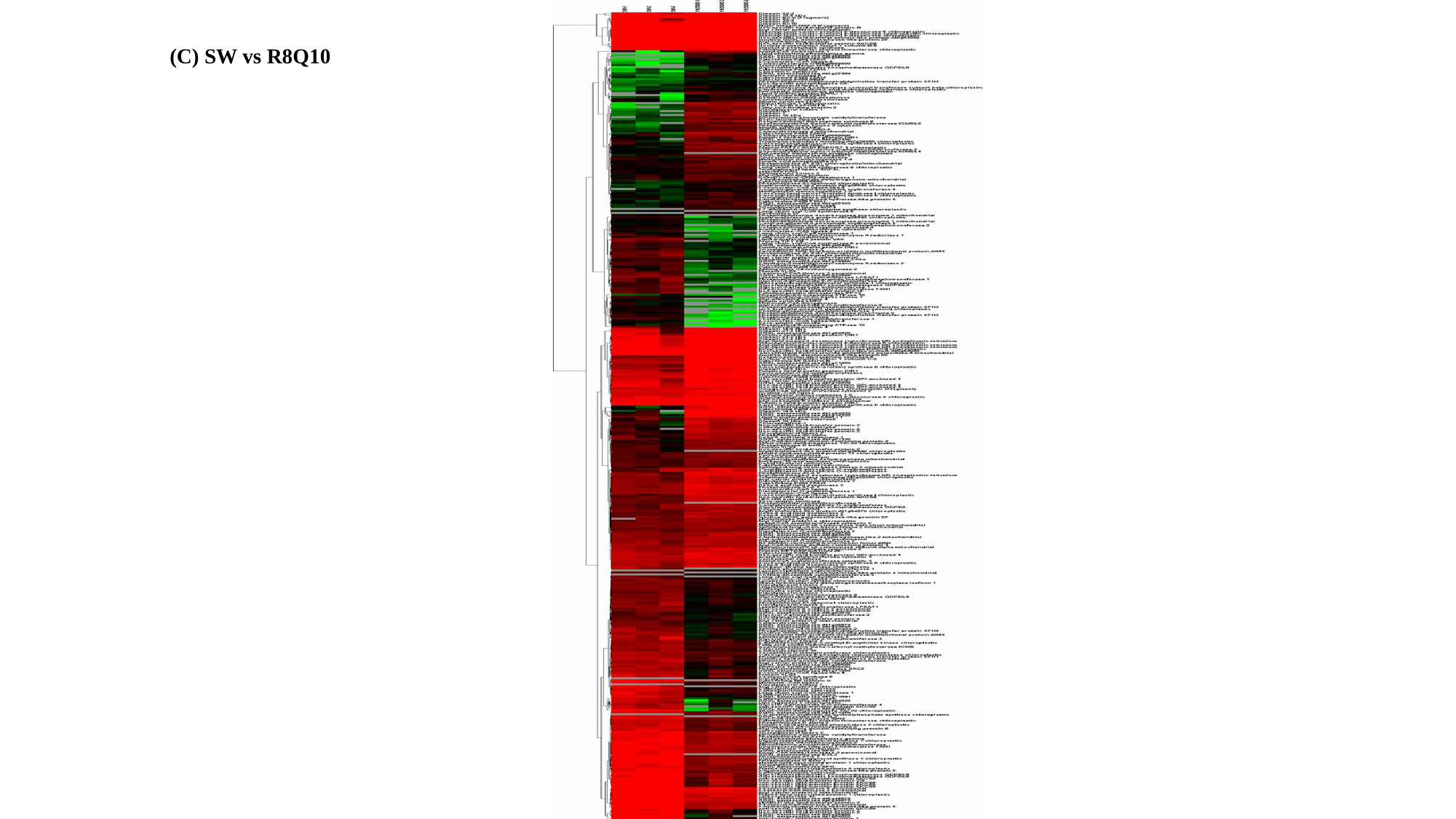

(C) SW vs R8Q10

## Slide 4
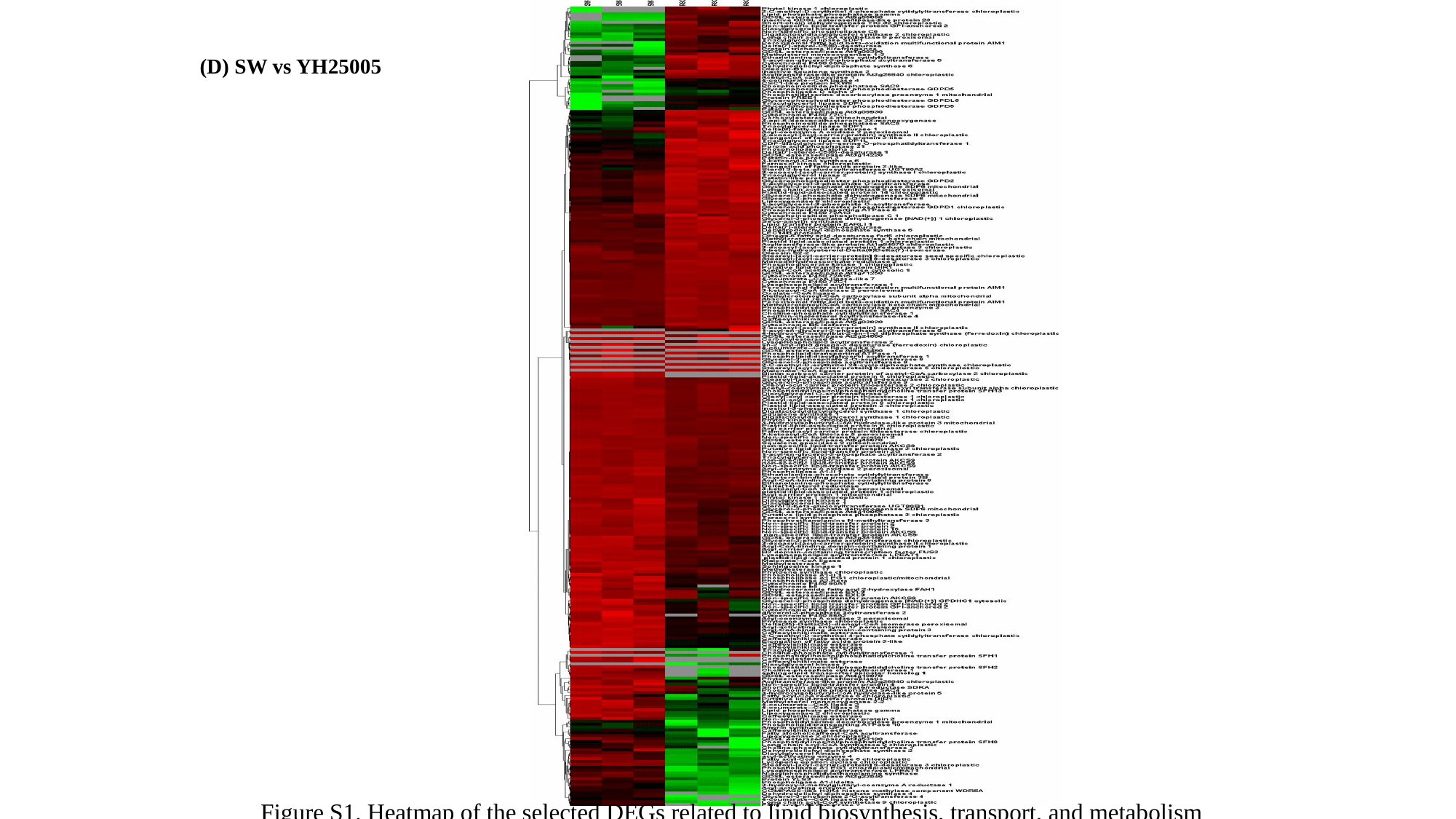

(D) SW vs YH25005
Figure S1. Heatmap of the selected DEGs related to lipid biosynthesis, transport, and metabolism
