# Supplementary material for: Comparative Transcriptomics Uncovers Upstream Factors Regulating BnFAD3 Expression and Affecting Linolenic Acid Biosynthesis in Yellow-Seeded Rapeseed (Brassica napus L.)
Source: Plants (Basel). 2024 Mar 7;13(6):760. doi: 10.3390/plants13060760 (PMC10974354; doi:10.3390/plants13060760)
Supplement: Supplementary file 1 [file plants-13-00760-s001.zip › Figure S2. Protein interaction network predicted based on DEGs.pptx]

## Slide 1
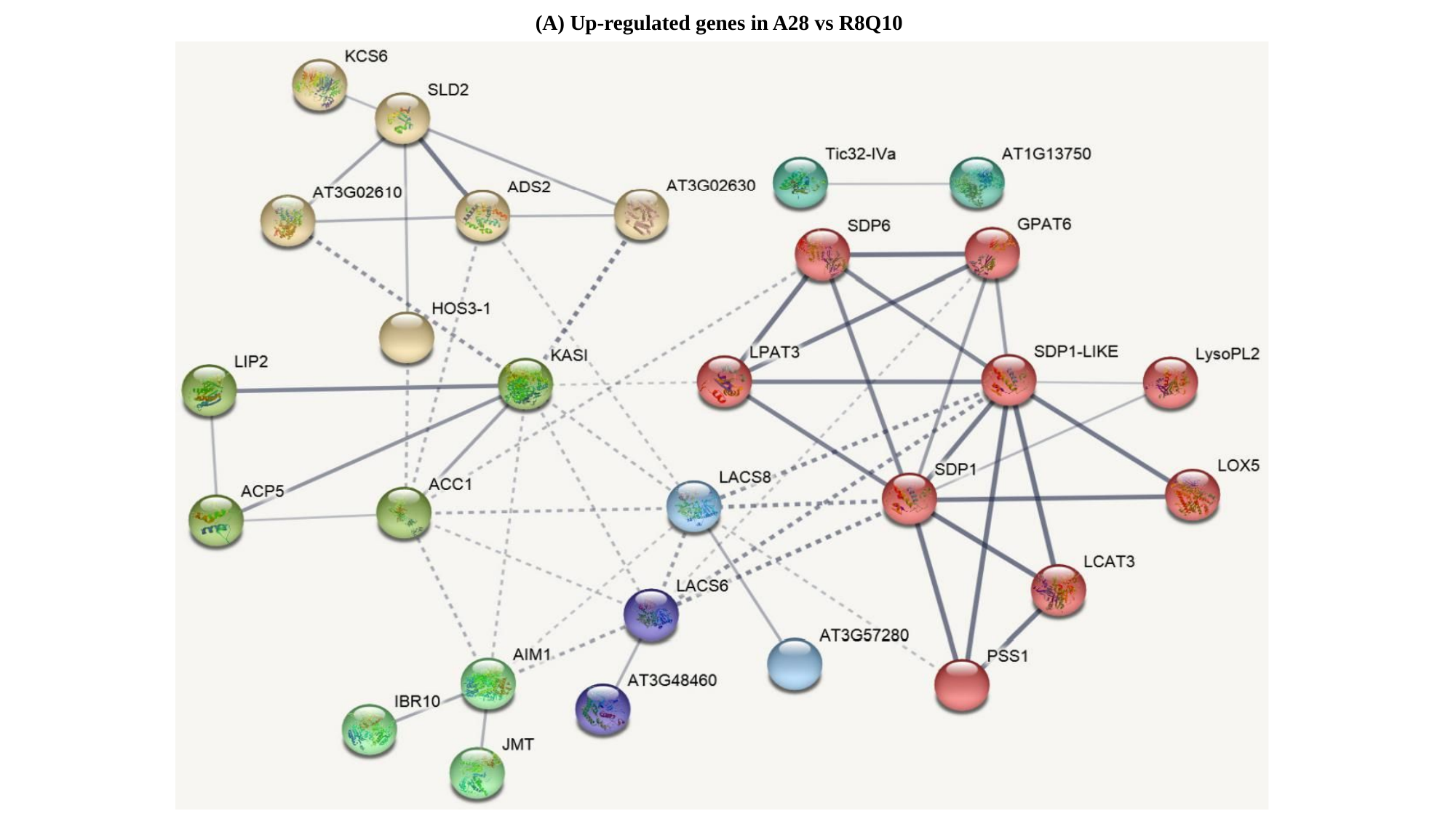

(A) Up-regulated genes in A28 vs R8Q10

## Slide 2
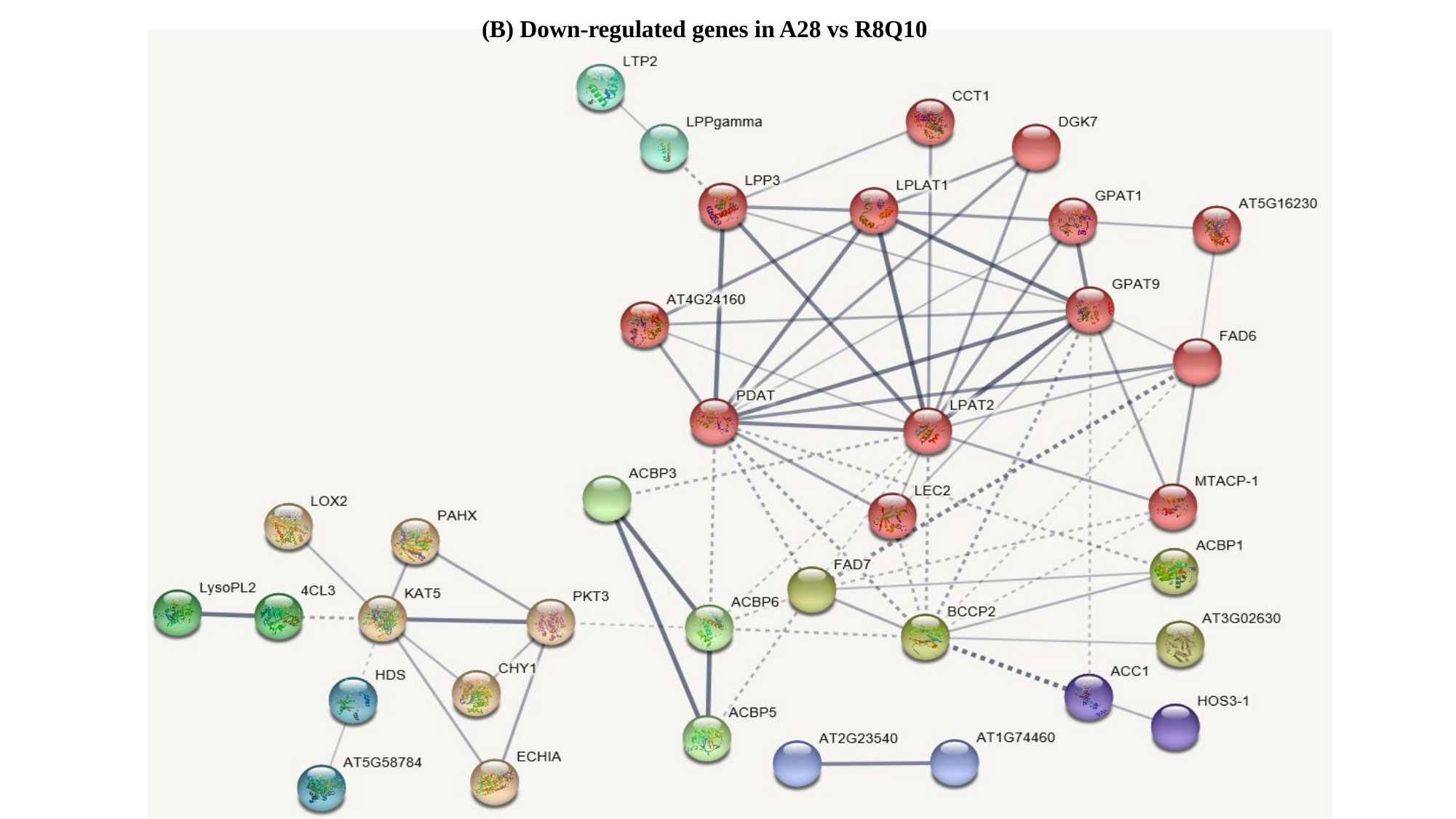

(B) Down-regulated genes in A28 vs R8Q10

## Slide 3
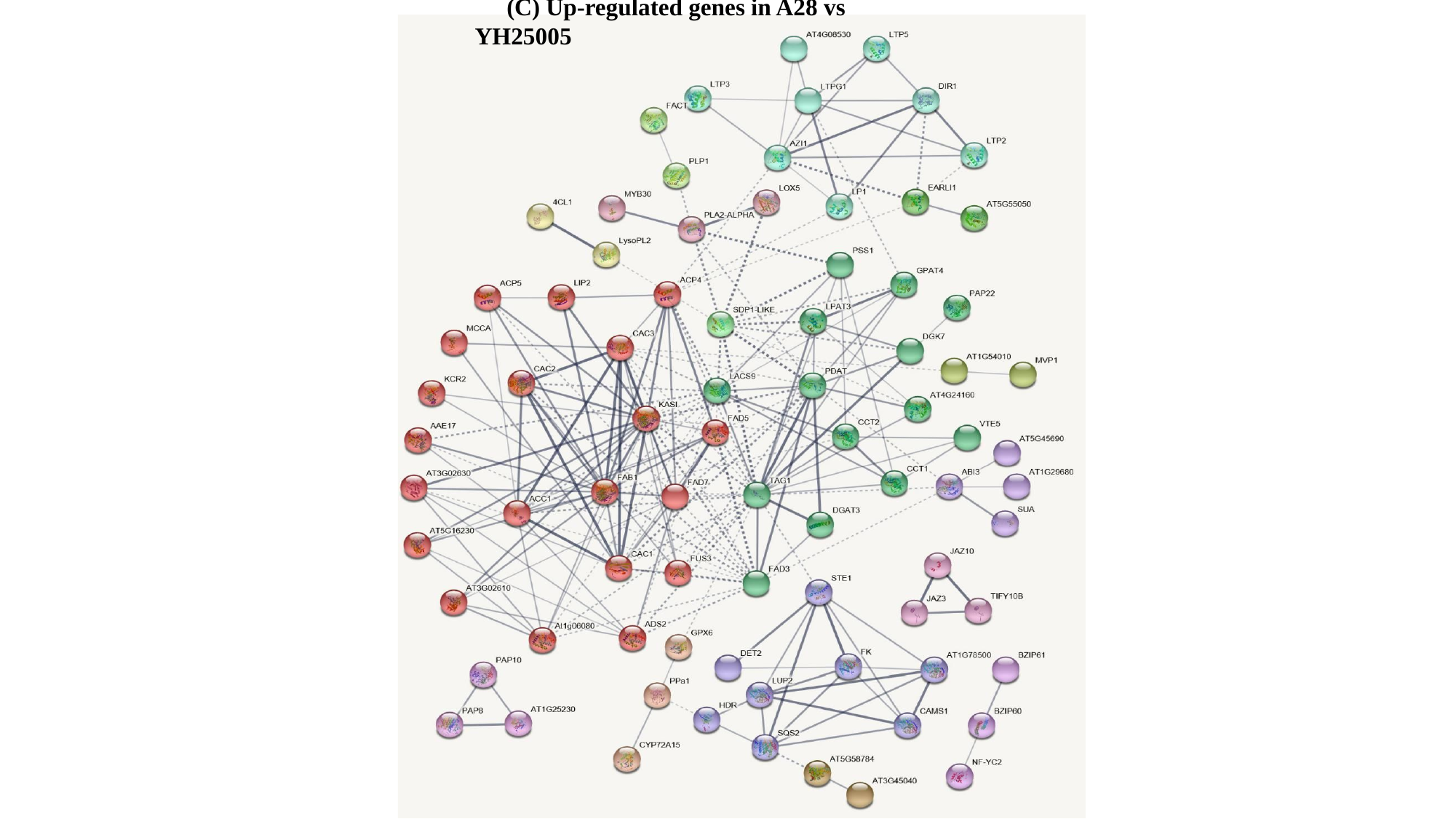

(C) Up-regulated genes in A28 vs YH25005

## Slide 4
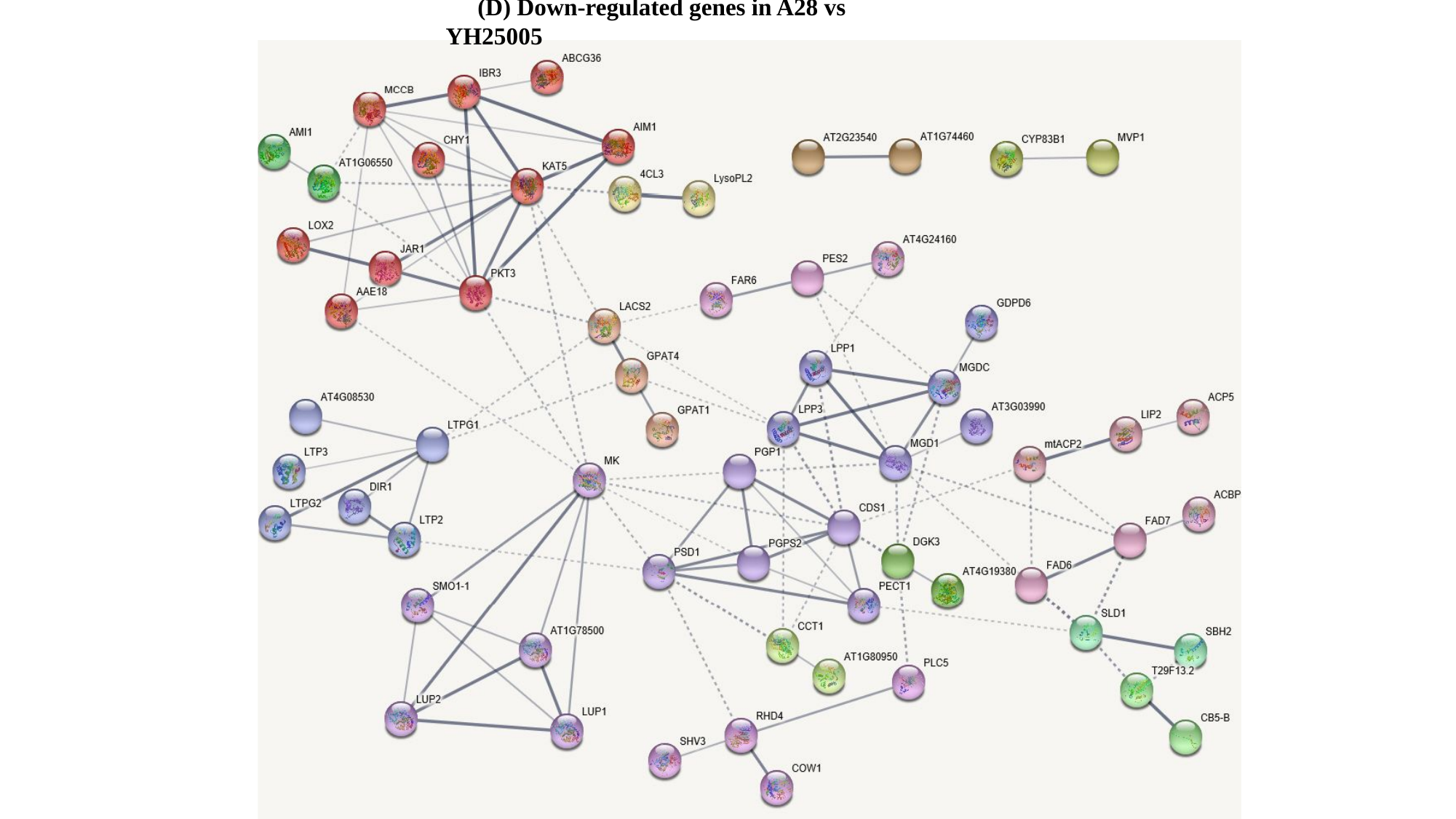

(D) Down-regulated genes in A28 vs YH25005

## Slide 5
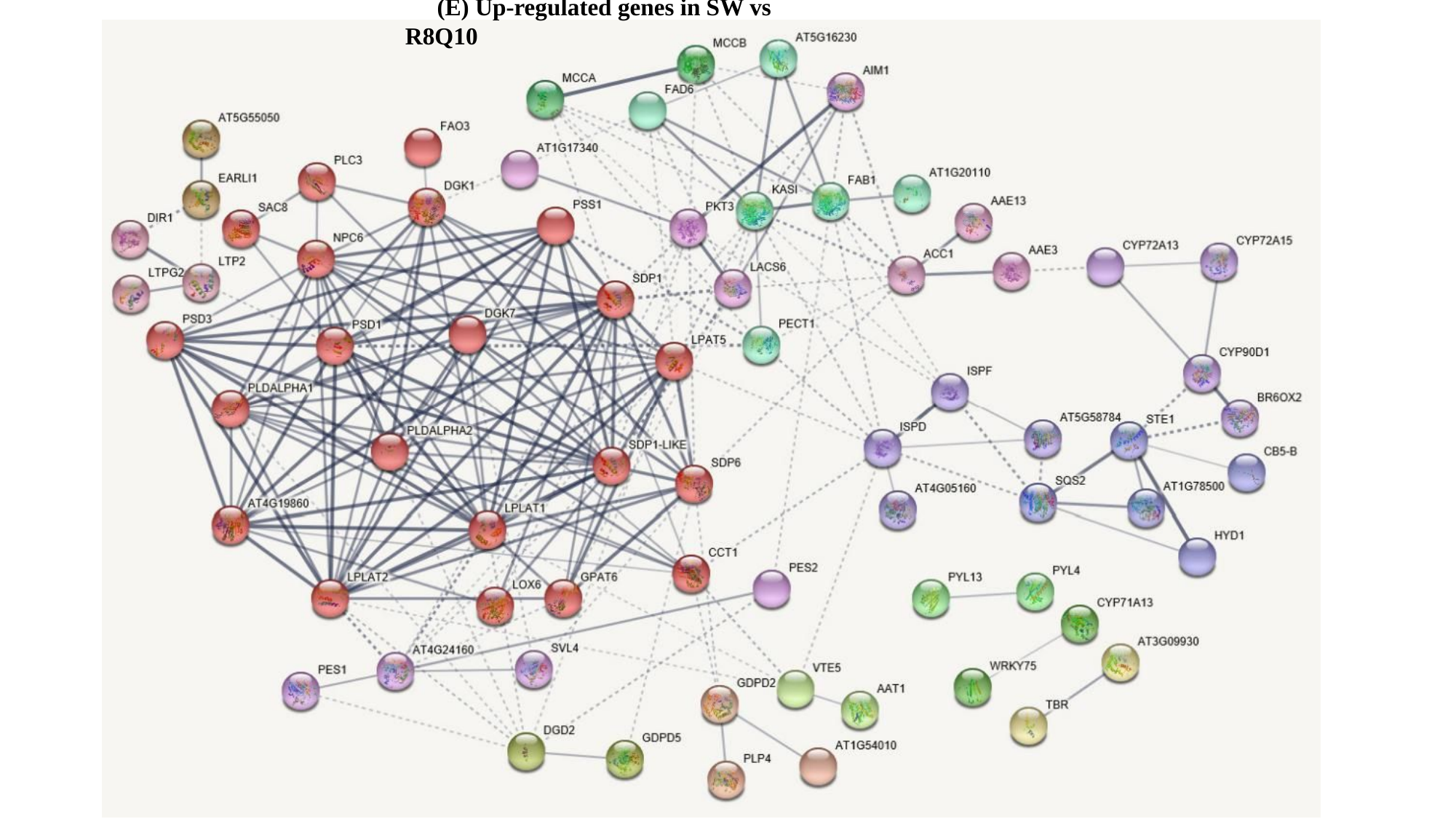

(E) Up-regulated genes in SW vs R8Q10

## Slide 6
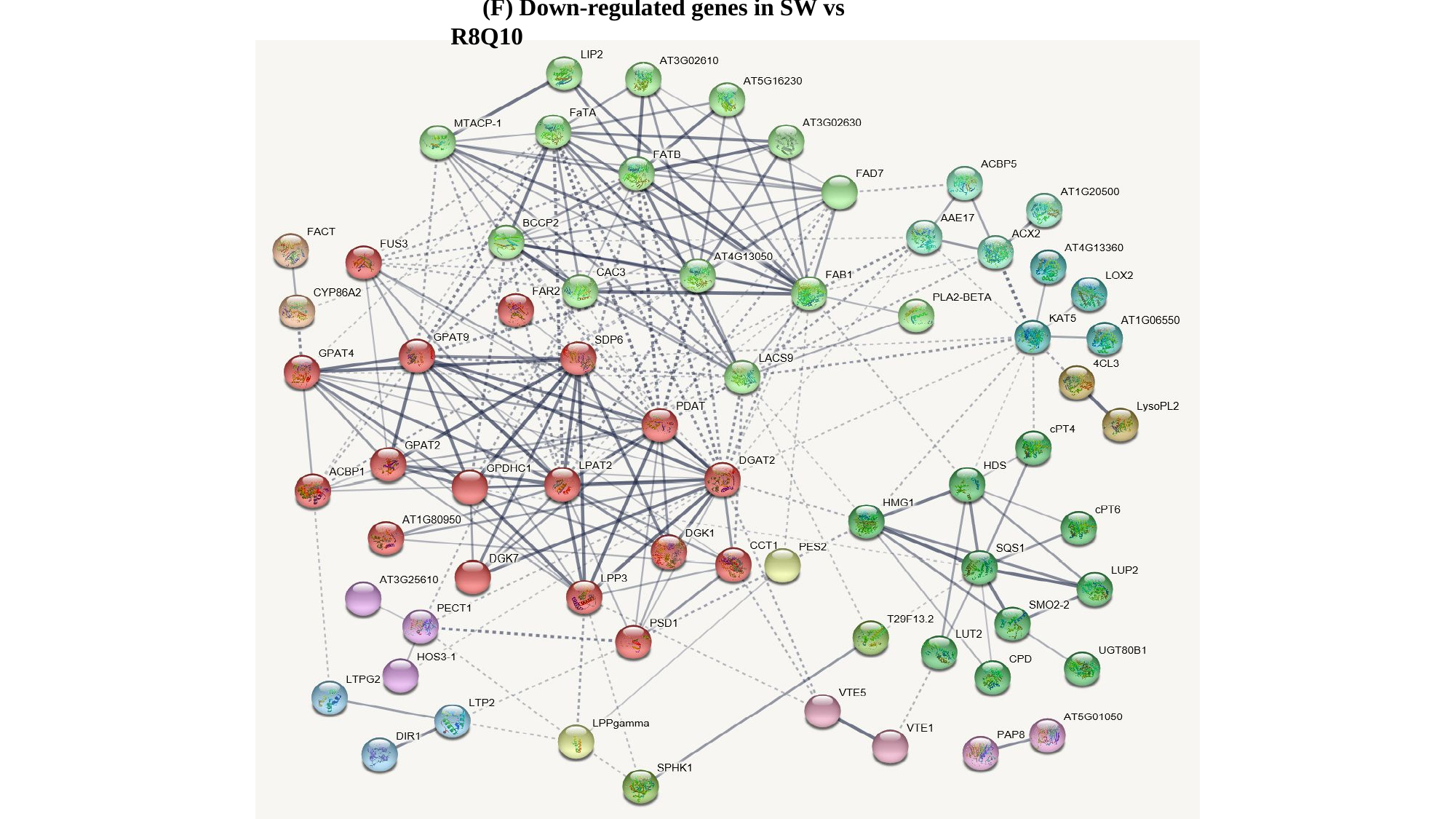

(F) Down-regulated genes in SW vs R8Q10

## Slide 7
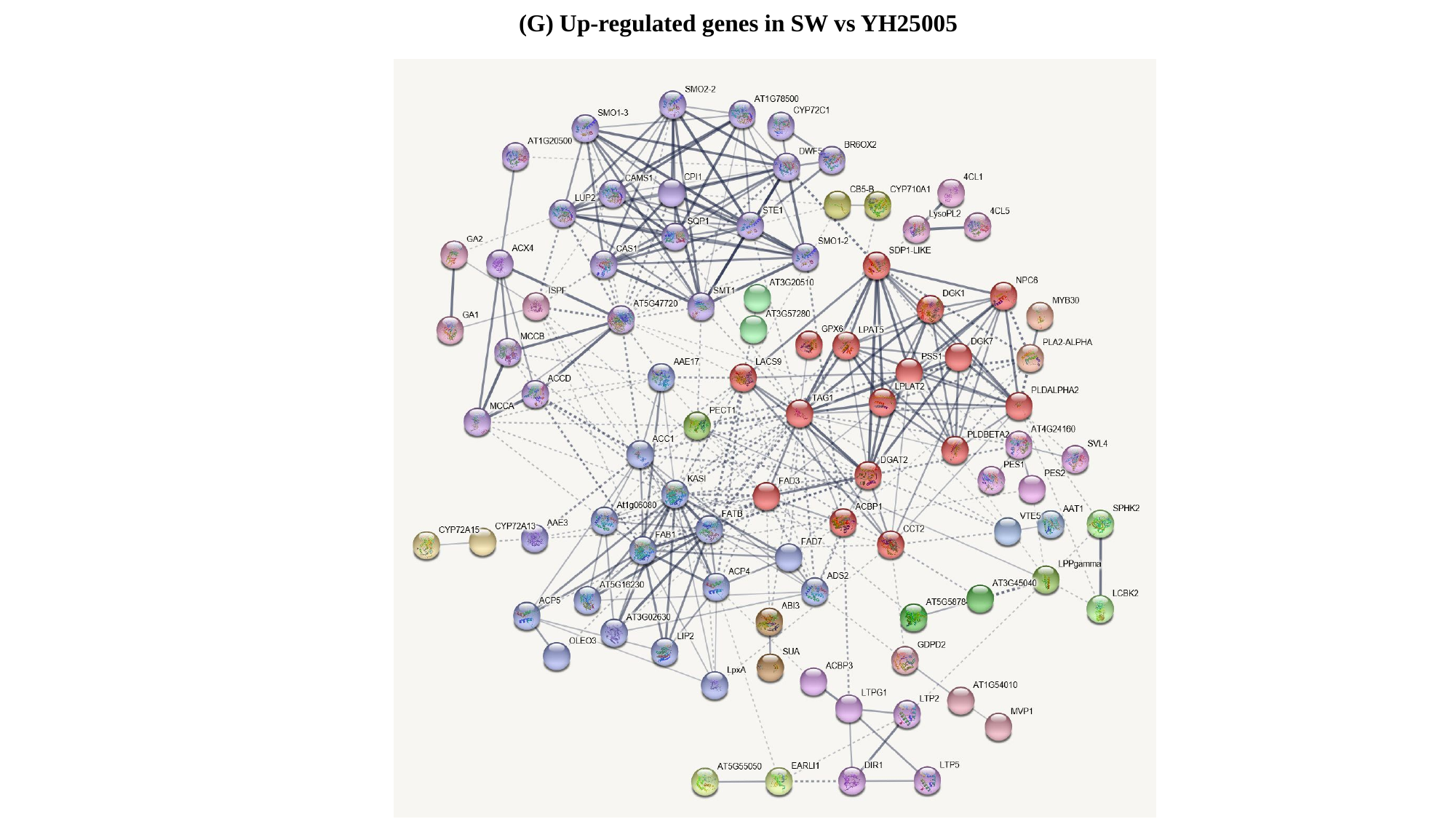

(G) Up-regulated genes in SW vs YH25005

## Slide 8
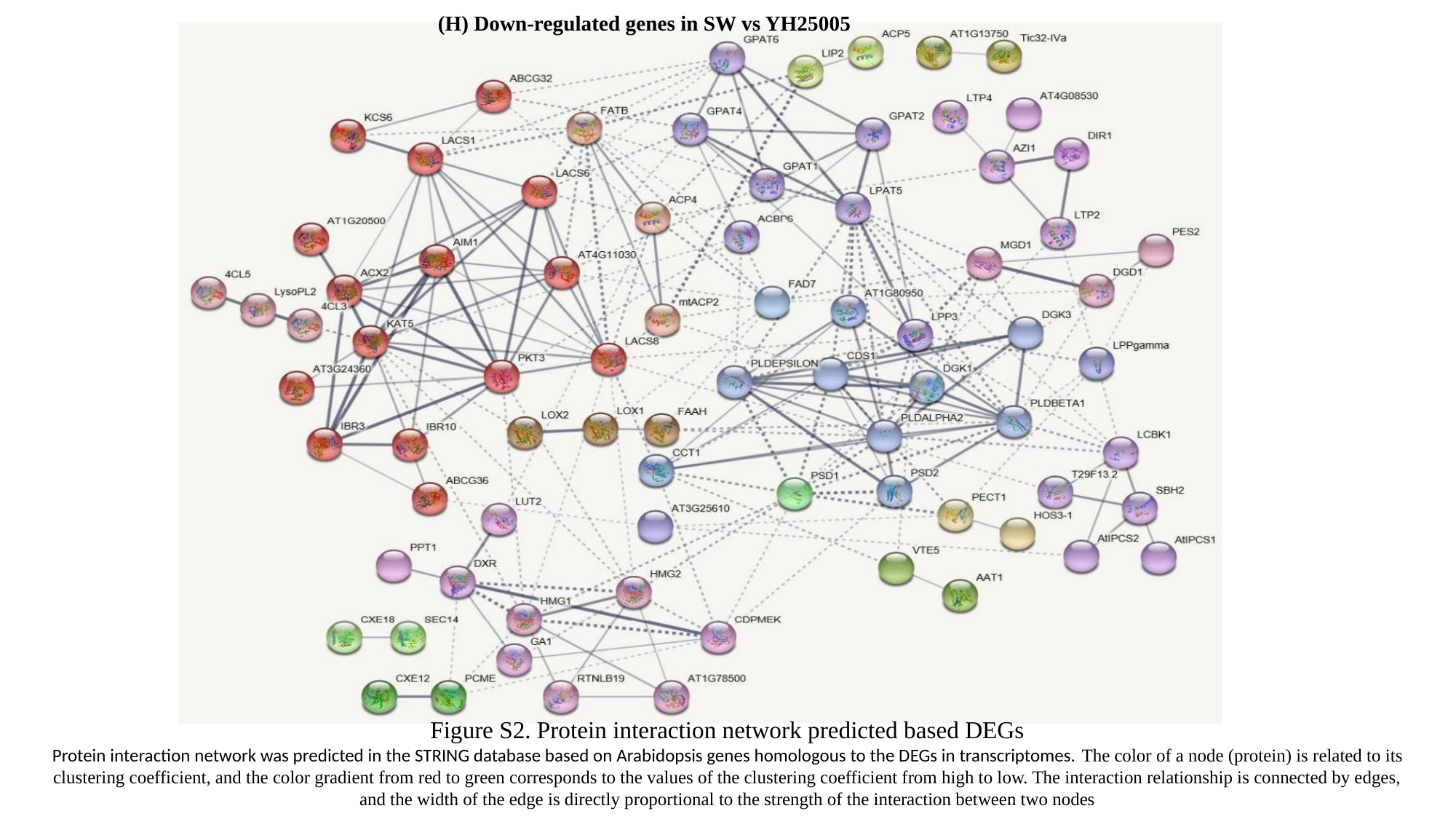

(H) Down-regulated genes in SW vs YH25005
Figure S2. Protein interaction network predicted based DEGs
Protein interaction network was predicted in the STRING database based on Arabidopsis genes homologous to the DEGs in transcriptomes. The color of a node (protein) is related to its clustering coefficient, and the color gradient from red to green corresponds to the values of the clustering coefficient from high to low. The interaction relationship is connected by edges, and the width of the edge is directly proportional to the strength of the interaction between two nodes
