# Supplementary material for: Comparative Transcriptomics Uncovers Upstream Factors Regulating BnFAD3 Expression and Affecting Linolenic Acid Biosynthesis in Yellow-Seeded Rapeseed (Brassica napus L.)
Source: Plants (Basel). 2024 Mar 7;13(6):760. doi: 10.3390/plants13060760 (PMC10974354; doi:10.3390/plants13060760)
Supplement: Supplementary file 1 [file plants-13-00760-s001.zip › Table S1. The primer sequences designed for the rapeseed genes used in RT.pdf]

Table S1 The primer sequences designed for the rapeseed genes used in RT-qPCR.

Rapeseed gene	Forward prime	Reverse prime
Actin7	CGCGCCTAGCAGCATGAA	GTTGGAAAGTGCTGAGAGATGCA
FAD2	CCAGCTCAAGAAACATGG	TACTTCCAGGAGAAGTAAGG
FAD3	CCTTGGTACAGAGGCAAG	ACCAAGTGATAGTGAGGG
FAD6	GCAGACAGCTATGGATTCAA	TCATCAATCTCAAACACCTCT
FAD7	ATCCAGACAGCGACTTGTT	GGCAAAGGTCCAGACTTATC
TT8	ACTTCTCATGTCACAACCCAC	ATCCATTGCGACGACGAAG
TT1	CTACAACAATCTTCAGATGC	CAACGCAGCAGTAACAAGGG
TT2	GTTAAACAGAGGAGCTTGG	GCCTGGTCTCAAGTAGTTC
TT12	GTCATGCTATGTTTGGAGAT	GCATATCCCAGTTCAAGTAATACA
TT16	TGCTCACATCGGTCTCATC	GAGCTCAAGCTTACATGTCTC
TTG1	TCCACCATCATCTACGAGAG	CGCAATGCCAATCCAAT
LODX	GAAGTACGCAAACGATCAAG	GAAGGTCAAAGCGCTTACAT
BAN	TTAACCGATGAAGGGAGTTT	GGTTTGATCATGTCTTTCTC
FLS	CTCAAGATTACAGGGAAGTGA	AATAGCCTCACGACGTAAAC
DFR	GAAGATGACAGGATGGATGT	GATCACCAATGTCGGGATAA
CHI	GAAATCGTCACAGGTTCGTT	TCAGAGTCTGTGTAAATCCC
CHS	AAGCGCATGTGCGATAAG	CCTAGCTTAGGGACTTCAAC
TT10	GCATTTGCTAAAGCACTCTC	ATGGAACTTCCTTAACCGTG
FUS3	TACTCCCCAAGAAAGCTGCG	TGTTGTTTGGCCAGTACCTGT
LEC2	GGGTCACTTGGGAGGATTG	TGTTATTGGACCAGTACTTGTAT
LEC1	ACAAGAACAAGAGTATCGTGC	AAACCATTTCCTCCAAAGGC
ABI3	ATTGACGACCTCGAGACCT	TATTCAAAGCCCTTTCCACG
NF-YC2	TGGTGTTGTGTGTGTTTAGA	ATTCCACTCTGATAAGCACG
bZIP25	CGTGTGTCTACTGTGGTG	TGTCAAATGAGGGAGCATC
bZIP10	AGAACAAGCTTGAGACTGAG	ACCAAAGAGCCTTGAGTAAG
bZIP67	AAAGAAGGCAAGAGGTGATG	TAATCTACCACCCAGCACTA
L1L	TGAGCTCGTGGCTTTGAA	ATCAAAAGCTTCCCTGGATT
